# **RETRACTION:** TMEM107 Inhibits EMT and Invasion of NSCLC Through Regulating the Hedgehog Pathway

**DOI:** 10.1111/1759-7714.70032

**Published:** 2025-03-07

## Abstract

**RETRACTION:** XuH. , DunS. , GaoY. , MingJ. , HuiL. , and QiuX. , “TMEM107 Inhibits EMT and Invasion of NSCLC Through Regulating the Hedgehog Pathway,” Thoracic Cancer 12, no. 1 (2021): 79–89, 10.1111/1759-7714.13715.33124203 PMC7779196

The above article, published online on 29 October 2020 in Wiley Online Library (wileyonlinelibrary.com), has been retracted by agreement between the authors; the journal Editor‐in‐Chief, Tateaki Naito; and John Wiley & Sons Australia, Ltd. The retraction has been agreed following concerns raised by a third party about duplicated images in Figures 3i and 4e. The authors acknowledge this error and note that Figure 4e inadvertently included images from the si‐TMEM07 and pNC groups of Figure 3i for the A549 cells. Therefore, the article must be retracted.

**RETRACTION:** H. Xu , S. Dun , Y. Gao , J. Ming , L. Hui , and X. Qiu , “TMEM107 Inhibits EMT and Invasion of NSCLC Through Regulating the Hedgehog Pathway,” Thoracic Cancer 12, no. 1 (2021): 79–89, 10.1111/1759-7714.13715.33124203 PMC7779196

The above article, published online on 29 October 2020 in Wiley Online Library (wileyonlinelibrary.com), has been retracted by agreement between the authors; the journal Editor‐in‐Chief, Tateaki Naito; and John Wiley & Sons Australia, Ltd. The retraction has been agreed following concerns raised by a third party about duplicated images in Figures 3i and 4e. The authors acknowledge this error and note that Figure 4e inadvertently included images from the si‐TMEM07 and pNC groups of Figure 3i for the A549 cells. Therefore, the article must be retracted.

